# Non-isolated tetralogy of fallot (TOF+): exome sequencing efficacy and phenotypic expansions

**DOI:** 10.1038/s41431-025-01916-8

**Published:** 2025-08-12

**Authors:** Julia Volpi, Xiaonan Zhao, Nichole Owen, Tia Evans, Muriel Holder-Espinasse, Nayana Lahiri, Eleanor Sherlock, Gemma Poke, Jeroen Breckpot, Koen Devriendt, Bjorn Cools, Alfredo Brusco, Giovanni Battista Ferrero, Enrico Grosso, Pradeep Vasudevan, Sara Loddo, Antonio Novelli, Maria Cristina Digilio, Aafke Engwerda, Marrit Hitzert, Alison Male, Lucy Bownass, Ruth Newbury-Ecob, Zosia Miedzybrodzka, Ruth Armstrong, Sally Ann Lynch, Gunnar Houge, Shiyi Xiong, Seema R. Lalani, Jill A. Rosenfeld, Pamela N. Luna, Chad A. Shaw, Daryl A. Scott

**Affiliations:** 1 Department of Molecular and Human Genetics, Baylor College of Medicine, Houston, TX 77030 USA; 2 Baylor Genetics Laboratories, Houston, TX 77030 USA; 3 Clinical Genetics Department Guy’s Hospital, London, SE1 9RT United Kingdom; 4 St George’s University Hospital NHS Foundation Trust, London, United Kingdom; 5 City St. George’s University of London, Cardiovascular and Genomics Institute, London, United Kingdom; 6 Genetic Health Service New Zealand, Wellington, New Zealand; 7 Center for Human Genetics, University Hospitals Leuven, Leuven, Belgium; 8 Department of Cardiovascular Sciences, University of Leuven, Leuven, Belgium; 9 Department of Pediatric and Congenital Cardiology, University Hospitals Leuven, Leuven, Belgium; 10 Department of Neurosciences Rita Levi-Montalcini, University of Turin, Turin, 10126 Italy; 11 Medical Genetics Unit, Città della Salute e della Scienza University Hospital, 10126 Turin, Italy; 12 Department of Clinical and Biological Sciences, University of Turin, 10049 Orbassano, TO Italy; 13 University Hospitals of Leicester NHS Trust, Leicester Royal Infirmary, Leicester, LE1 5WW United Kingdom; 14 Translational Cytogenomics Research Unit, Bambino Gesù Children’s Hospital, IRCCS, Rome, Italy; 15 Medical Genetics Unit, Academic Department of Pediatrics, Bambino Gesù Children’s Hospital, IRCCS, Rome, Italy; 16 Department of Genetics, University of Groningen, University Medical Center Groningen, Groningen, The Netherlands; 17 North East Thames Regional Genetic Service, Great Ormond Street Hospital for Children NHS Foundation Trust, London, United Kingdom; 18 St Michael’s Hospital, Southwell St, Bristol, BS2 8EG United Kingdom; 19 School of Medicine, Medical Sciences, Nutrition and Dentistry, University of Aberdeen, Aberdeen, United Kingdom; 20 North of Scotland Clinical Genetics Service, NHS Grampian, Edinburgh, United Kingdom; 21 Department of Clinical Genetics, Cambridge University Hospitals NHS Foundation Trust, Cambridge, CB2 0QQ United Kingdom; 22 Children’s Health Ireland at Crumlin, Crumlin, Dublin 12, Republic of Ireland; 23 Department of Medical Genetics, Haukeland University Hospital, Bergen, Norway; 24 Prenatal Diagnosis Center & Fetal Medicine Unit, Shanghai Key Laboratory of Maternal Fetal Medicine, Shanghai, 200092 China; 25 Shanghai Institute of Maternal-Fetal Medicine and Gynecologic Oncology, Shanghai First Maternity and Infant Hospital, School of Medicine, Tongji University, Shanghai, 200092 China

**Keywords:** Genetics research, Genetic testing, Medical genetics, Disease genetics

## Abstract

Tetralogy of Fallot (TOF) is the most common cyanotic congenital heart defect (CHD). TOF may present in isolation or in conjunction with one or more non-cardiac congenital anomalies or neurodevelopmental disorders (TOF+). Uncertainty regarding the efficacy of various genetic testing strategies, and an incomplete understanding of the genetic causes of TOF+, may lead to hesitancy in recommending genetic testing, particularly, clinical exome sequencing (cES). Here, we analyzed cES data from 131 individuals with TOF+. A definitive or probable diagnosis was made for 31 individuals, yielding a diagnostic rate of 23.6% (31/131). One individual received three diagnoses. Commercially available CHD panels would have detected only 27.3% (9/33) to 63.6% (21/33) of the diagnoses made by cES. We then used a machine learning approach to identify four genes for which there is sufficient evidence to support a phenotypic expansion including TOF: *DVL3, MED13L, PUF60*, and *MEIS2*. Since chromosomal microarray analysis (CMA) has been reported to have a diagnostic efficacy of 10–20% in individuals with TOF, we conclude that cES should be considered for all individuals with TOF+ for whom a molecular diagnosis has not been established by CMA. We also conclude that TOF represents a low penetrance phenotype associated with genetic syndromes caused by pathogenic variants in *DVL3, MED13L, PUF60*, and *MEIS2*.

## Introduction

Congenital heart defects (CHDs) affect 9.1 per 1000 live births. Tetralogy of Fallot (TOF) is the most common cyanotic CHD with an incidence of 0.34 per 1000 live births [1]. TOF is a well-characterized congenital cardiac abnormality caused by an anterior and cephalad deviation of the muscular outlet of the ventricular septum leading to an anteriorly malaligned ventricular septal defect (VSD), an overriding aorta, right ventricular outflow tract obstruction, and secondary right ventricular hypertrophy [2]. These defects lead to obstruction of proper blood flow and low systemic oxygen levels. Affected individuals require surgery in infancy [3]. TOF exists along a spectrum with TOF with absent pulmonary valve (TOF-APV) and TOF with pulmonary atresia (TOF-PA) at the severe end [4]. In TOF-APV, the absence of a pulmonary valve results in unique challenges due to the combination of pulmonary annular stenosis, severe pulmonary regurgitation, and airway compression secondary to aneurysmal dilatation of the pulmonary arteries [5]. In TOF-PA, flow into the pulmonary arteries is blocked. This can exist with or without major aortopulmonary collateral arteries (MAPCAs) in which pulmonary blood flow is supplied by collateral vessels from the systemic circulation [6]. TOF-PA and TOF-PA-MAPCA are frequently associated with 22q11.2 deletion syndrome [7–9].

TOF may present in isolation or in conjunction with one or more non-cardiac congenital anomalies or neurodevelopmental disorders (TOF + ). In some cases, TOF and TOF+ have identifiable genetic etiologies. For example, isolated TOF can be caused by pathogenic variants in *NOTCH1*, *TBX1, FLT4*, or *GATA4* [10, 11]. TOF is also the most common CHD associated with 22q11.2 deletion syndrome and is an established phenotype for individuals with many genetic disorders including Alagille syndrome, CHARGE syndrome, and Kabuki syndrome [7, 12]. TOF can also recur in families even without a known genetic cause and can be associated with environmental factors including maternal alcohol use and maternal rubella [13, 14].

Identifying a molecular cause for TOF+ can facilitate accurate risk assessments and aid in medical management decisions. However, questions regarding the efficacy of various genetic testing strategies, and an incomplete understanding of genes known to cause TOF+, may cause health care professionals to hesitate in recommending genetic testing for affected individuals.

Here we use data from 131 individuals with TOF+ to determine the diagnostic yield of clinical exome sequencing (cES), compare the efficacy of CHD gene panels to cES, and identify phenotypic expansions involving TOF using molecular and clinical data form this cohort and the DECIPHER database [15, 16].

## Methods

### Human subjects research

This work was approved by the institutional review board of Baylor College of Medicine (Protocol H-47546) and was conducted in accordance with the ethical standards of this institution’s committee on human research and international standards.

We searched the Baylor Genetics (BG) clinical database for individuals with TOF listed in their indication who were referred for cES from January 2012 – September 2023. Individuals who received a molecular diagnosis based on a test other than cES (i.e., chromosome analysis, chromosomal microarray analysis (CMA), or gene panel testing) were excluded from this cohort. We identified 130 individuals with a personal history of TOF+ and one individual with a family history of TOF+ in an identical twin. Of these 131 individuals, 71 (BG1-BG71) had a sequence variant(s) reported back to their physicians as being possibly associated with one or more of the phenotypes listed in their indication for testing.

Additionally, we searched the DECIPHER database for individuals with a diagnosis of TOF in association with a sequence variant(s) or a copy number variant(s) that were less than 1.5 Mb in size and affected less than 20 protein-coding genes [15, 16]. We then contacted representatives of the organization that oversaw their deposit into the database and received permission to include 14 individuals in our study (D1-D14).

Molecular and clinical data for individuals BG1-BG71 and D1-D14 are summarized in Supplemental Tables S1 and S2.

### Reclassification of sequence and copy number variants

Variants were reclassified by laboratory geneticists as pathogenic, likely pathogenic, a variant of uncertain significance (VUS), likely benign, or benign, based on the 2015 American College of Medical Genetics and Genomics (ACMG) standards for the interpretation of sequence variants (completed in April 2024) or the 2020 American College of Medical Genetics and Genomics (ACMG) and the Clinical Genome Resource (ClinGen) technical standards for the interpretation and reporting of constitutional copy number variants by clinical laboratory geneticists (completed in October 2024) [17, 18].

### Determination of diagnostic certainty

The molecular and clinical data from subjects BG1-BG71 and D1-D9 were reviewed to categorize the diagnostic certainty associated with the variants reported back to physicians. Briefly, all diagnoses were binned into “definitive”, “probable”, or “provisional” categories based on criteria previously outlined by Scott et al. with the following additions: 1) An individual with a pathogenic variant (P) and a VUS in trans in a gene with an associated autosomal recessive condition, and phenotypic data suggestive of the disorder, was considered to have a probable diagnosis, and 2) an individual with a likely pathogenic variant (LP) and a VUS in trans in a gene with an associated autosomal recessive condition, and phenotypic data suggestive of the disorder, was considered to have a provisional diagnosis [19].

### Clinical exome sequencing efficacy

The efficacy of cES was calculated by summing the number of BG cases with a definitive or probable diagnosis and dividing by the total number of BG cases. For individuals with multiple variants, and thus more than one possible diagnosis, we used each individual’s variant of highest diagnostic certainty when calculating efficacy.

### Coverage of commercially available CHD gene panels

To evaluate the coverage of commercially available CHD gene panels, we identified the genes included in four commercially available tests whose descriptive labels were “Congenital Heart Disease Panel,” “Congenital Structural Heart Disease Panel,” “Comprehensive Congenital Heart Disease Panel,” and “Congenital Heart Defect NGS Panel” (completed in March 2025; Supplemental Table S4). We compared the gene coverage of these panels to the genes affected in our cES cohort.

### Literature and database searches

We searched the OMIM database (https://www.omim.org/) and the scientific literature for reports in which TOF candidate genes, or their associated genetic disorder(s), were associated with TOF [20]. We searched the Mouse Genome Informatics database (MGI; http://www.informatics.jax.org/) and the scientific literature to determine if the mouse homologs of TOF candidate genes were associated with the development of CHD [21].

### Generating TOF-specific rank annotation scores using machine learning

We used a previously published machine learning algorithm to determine the similarity between all RefSeq genes and genes known to cause TOF [22, 23]. Briefly, this algorithm integrates annotation data from various genome-scale knowledge sources to construct a pattern in genomic feature space and then ranks all RefSeq genes with respect to their similarity to a set of training genes associated with a specific phenotype using quantitative similarity metrics [24–30].

We then trained our machine learning algorithm with 53 genes that have been clearly shown to cause TOF in humans: *ALDH1A2, CACNA1C, CFC1, CHD4, CHD7, CITED2, CXCR4, DOCK6, EP300, FGF8, FLNA, FLT4, FOXC1, FOXC2, FOXH1, GATA4, GATA5, GATA6, GDF1, HAND1, HAND2, ISL1, JAG1, KDM6A, KDR, KMT2C, KMT2D, KRAS, MESP1, MKKS, NAA15, NFATC1, NKX2-5, NKX2-6, NODAL, NOTCH1, NOTCH2, PITX2, PTPN11, RAF1, RBM10, ROBO1, SALL1, SMAD4, SMARCC2, SOS1, TBX1, TBX2, TBX5, TBX20, TDGF1, WASHC5*, and *ZFPM2*. These genes were selected for the training gene set based on the strength of the data in support of their TOF association as demonstrated in reviews, OMIM, StatPearls, and/or multiple case reports [20, 31–40]. Hence, it does not represent a comprehensive list of all genes with reported associations to TOF.

**Fig. 1:**
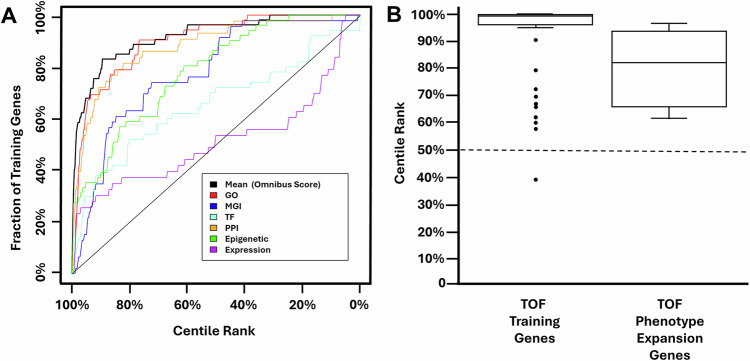
Generating and validating TOF-specific rank annotation scores for all RefSeq genes. **A** A previously published machine learning algorithm was trained using 53 genes known to cause TOF in humans. Receiver operating characteristic (ROC) style curves were generated based on a leave-one-out cross-validation study analysis performed for each knowledge source (colored lines). The area under the omnibus curve (black) indicates the ability of the algorithm to identify genes in the training set more effectively than chance (diagonal black line). **B** Box plots showing the algorithmically generated TOF-specific rank annotation scores for the TOF training genes and the four candidate genes, *DVL3*, *MED13L*, *PUF60*, and *MEIS2*, for which there was sufficient evidence to support a phenotypic expansion involving TOF (Table 1). The median rank annotation scores of these groups—99.6% and 82.5%, respectively—were greater than what would be expected by chance alone (50%; dotted line).

Leave-one-out cross-validation studies were used to test the performance of the algorithm [41]. Receiver operating characteristic (ROC) style curves were generated based on leave-one-out validation analyses to represent the algorithm’s effectiveness (Fig. 1A). The area under the curve (AUC) and above the diagonal line, which represents the result that would be generated by chance alone, represents the effectiveness of the algorithm. An omnibus curve produced using fit data from all knowledge sources had a positive AUC, indicating that the algorithm could distinguish between the TOF genes in the training set and all other RefSeq genes at a rate greater than random chance.

Using the full 53 gene training set, we then generated TOF-specific rank annotations scores for all RefSeq genes (Supplemental Table S3). By definition, these scores ranged from 0–100% with a median score of 50%. The TOF-specific pathogenicity score of the 53 genes in the training set range from 38.9–100%, with a median score of 99.6% (Fig. 1B). The following genes were outliers: *CHD4* (38.9%), *WASHC5* (39.2%), *KDM6A* (57.6%), *RBM10* (60.2%), *SMARCC2* (61.9%), *NAA15* (65.7%), *DOCK6* (66.9), *KMT2C* (69.5%), *GDF1* (72.5%), *KMT2D* (79.3%), and *TBX2* (90.7%), meaning that their TOF-specific rank annotation scores fell below 1.5 times the interquartile range (IQR) from the lower quartile for this gene set.

## Results

### Diagnostic yield of cES

Of the 131 individuals identified with TOF in our cohort, cES provided a definitive diagnosis for 18.3% (24/131) and a probable diagnosis for 5.3% (7/131), yielding a molecular diagnostic efficacy for cES of 23.7% (31/131). One individual (BG64) received three diagnoses. A provisional diagnosis was made in an additional 30.5% (40/131) of our cohort.

### Coverage of commercially available CHD gene panels

Of the 33 definitive or probable diagnoses made in our cohort, between 27.3% (9/33) and 63.6% (21/33) could have been made using one of four commercially available CHD gene panels based on gene coverage. These diagnoses involved 30 unique genes. Between 20% (6/30) and 60% (18/30) of these genes were covered in one of the four commercially available CHD gene panels (Supplemental Table S4).

### Phenotypic expansions involving TOF

A subset of individuals in our BG and DECIPHER cohort carried pathogenic or likely pathogenic sequence variants in genes clearly associated with the development of TOF. These genes included *BRAF*, *CDK13*, *CHD4*, *CHD7*, *DOCK6*, *EP300*, *FLT4*, *FOXC2*, *JAG1*, *KMT2D*, *NAA15*, *NOTCH1*, *RBM10*, *SMAD4*, *SMARCA4*, and *WASHC5* (Table S1). Genes harboring sequence variants associated with a definitive or probable diagnosis, and were not known to cause TOF, were designated as TOF candidate genes.

To determine which of the TOF candidate genes were likely to be contributing to the development of TOF, we considered 1) whether the gene’s machine learning generated TOF-specific rank annotation score was positive ( > 50%) or high ( ≥ 85%), 2) the existence of previously published cases in which TOF was associated with variants affecting the gene, and 3) whether CHD had been observed in mice models involving the gene’s homolog. Among TOF candidate genes associated with a definitive or probable diagnoses, our analysis revealed four genes for which there was sufficient evidence to support a phenotypic expansion involving TOF – *DVL3*, *MED13L*, *PUF60*, and *MEIS2*. As summarized in Table 1 and Fig. 1B, these genes had a median TOF-specific rank annotation score of 82.5%, all had previously been reported in association with case reports of TOF in the literature, and in the case of *DVL3* and *MEIS2*, their homologues were associated with the development of CHD in mouse models. Detailed descriptions of the evidence in support of *DVL3*, *MED13L*, *PUF60*, and *MEIS2*’s associations with TOF are presented in the Discussion.

**Table 1:** Genes for which there is sufficient evidence to support a phenotypic expansion involving TOF.

Gene	Disorder [MIM #]	Subject ID; Variant; ACMG Interpretation	# Individuals in Cohort; Diagnostic Certainty	TOF-specific rank annotation score	Other TOF cases reported in gene / syndrome?	CHD in Mice?	References
*DVL3*	Robinow syndrome, autosomal dominant 3; [616894]	**BG45**; c.1672_1705del [NM_004423.4], p.(Y558Tfs*99); Likely Pathogenic	1; Probable	96.8%	No / Yes	Yes	Bain et al. [49]; Etheridge et al. [50]
*MED13L*	*MED13L*-syndrome [616789]	**D2**; c.3191dup [NM_015335.5], p.(T1065Hfs*9); Pathogenic	1; Definitive	85.1%	Yes / Yes	No	Harvey et al. [55]
*PUF60*	Verheij syndrome [615583]	**BG61**; c.628C>T [NM_078480.3], p.(Q210*); Pathogenic **D7**; c.449_457del [NM_078480.3], p.(A150_F152del); Pathogenic **BG62**; c.1492_1494del [NM_078480.3], p.(I498del); VUS	3; Definitive, Definitive, Provisional	79.9%	Yes / Yes	No	El Chehadeh et al. [59]; Baum et al. [61]
*MEIS2*	*MEIS2*-related syndrome [600987]	**BG31**; c.777_781del [NM_170674.5], p.(A260Tfs*5); Likely Pathogenic **BG26**; c.1247 C > G [NM_170675.5], p.(P416R); VUS	2; Probable, Provisional	61.9%	Yes / Yes	Yes	Louw et al. [64]; Verheije et al. [66]; Chen et al. [65]

*VUS* variant of uncertain significance.

A similar process was used to evaluate the candidate CNVs found in D10-D14, considering the individual genes involved. However, none were found to have sufficient evidence to support a phenotypic expansion involving TOF.

## Discussion

### Diagnostic efficacy of cES in individuals with TOF+ and comparisons to CHD gene panels

cES has emerged as a powerful tool with the potential to provide a precise molecular diagnosis that informs clinical management and improves genetic counseling for affected individuals and their families [42, 43]. The evaluation of cES diagnostic efficacy is key in supporting the decision-making process for physicians and setting expectations for patients and their families.

In our TOF+ cohort, cES provided a definitive or probable diagnosis in 23.7% (31/131) of cases. In addition, a provisional diagnosis was made 30.5% (40/131) of our cohort, most of which were associated with a VUS which could be reclassified as likely pathogenic or pathogenic in the future.

The ability of cES to interrogate all disease-relevant genes, regardless of their known association with a specific phenotype, leads to an increased ability to make a molecular diagnosis in individuals with TOF when compared to a gene panel. Specifically, we found that of the 33 definitive or probable diagnoses made in our TOF cohort, only 27.3% (9/33) to 63.6% (21/33) could have been made using one of four commercially available CHD gene panels.

### Phenotypic expansions involving TOF

#### *DVL3*

Heterozygous gain-of-function variants resulting in a -1 frameshift of the last exon in *DVL3* are associated with Robinow syndrome, autosomal dominant type 3 (DRS3; MIM# 616894) [44]. Robinow syndrome is characterized by mesomelic limb shortening, genital hypoplasia, and distinctive facial features, and is associated with a variety of congenital anomalies. Cardiac defects associated with Robinow syndrome include right ventricular outflow obstructions such as pulmonary stenosis and, if present, are a major cause of morbidity and mortality [45]. *DVL3* is one of three disheveled genes (*DVL1*, *DVL2*, and *DVL3*) which are all early mediators of the Wnt signaling pathway and promote transition of undifferentiated mesoderm cells to cardiac lineages [46]. Wnt signaling has been implicated in myocardial specification, cardiac morphogenesis, and cardiac valve formation and is essential for development of cardiac neural crest cells and the outflow tract itself [47, 48]. All variants known to cause autosomal dominant Robinow syndrome occur in genes within the Wnt signaling pathway (*DVL1*, *DVL3*, or *WNT5A*). In our cohort, BG45 had a probable diagnosis of Robinow syndrome caused by *DVL3*. One other individual with TOF and Robinow syndrome has been described in the literature, but the causative gene was not reported [49]. *Dvl3*-null mice die perinatally with cardiac outflow tract abnormalities, including double outlet right ventricle (DORV) and persistent truncus arteriosus [50]. The pathogenesis of TOF and DORV have overlapping genetic causes and similarly result from disturbances of the second heart field [51]. These findings, in combination with *DVL3*’s high TOF-specific rank annotation score (96.8%), suggest that individuals with DRS3 may present with TOF.

#### *MED13L*

Heterozygous loss-of-function variants in *MED13L* are associated with *MED13L* syndrome (MIM# 616789) [52]. *MED13L* syndrome is characterized by delayed psychomotor development, poor speech acquisition, and distinctive facial differences, including frontal bossing, upslanting palpebral fissures, depressed nasal bridge, a bulbous tip, and macrostomia. The condition has variable expressivity of cardiac defects, including dextro-looped transposition of the great arteries and VSD [53, 54]. In our cohort, D4 had TOF and definitive diagnosis of *MED13L* syndrome. Two additional individuals with TOF and *MED13L* variants have been described; one with a de novo p.(Ser2131Leu) variant and one with a de novo 115 kb out of frame deletion of *MED13L* exons 3-4 [53, 55]. Data from the International Mouse Phenotyping Consortium (IMPC) suggest that male *Med13l*-null mice may have an increased incidence of abnormal blood vessel morphology compared to controls [56]. These findings, and *MED13L*’s high TOF-specific rank annotation score (85.1%), suggest that individuals with *MED13L* syndrome can present with TOF.

#### *PUF60*

Heterozygous pathogenic variants in *PUF60* are the cause of Verheij syndrome (MIM# 615583), and *PUF60* haploinsufficiency plays a role in 8q24.3 deletion syndrome [57, 58]. Verheij syndrome is characterized by delayed psychomotor development, growth disturbance, microcephaly, vertebral skeletal anomalies, and facial differences including long philtrum, short nose, thin upper lip, and short neck. Additional phenotypes include coloboma, renal anomalies, and cardiac defects such as truncus arteriosus and VSD [59]. In our cohort, we had three individuals with TOF who carried *PUF60* variants. BG61 and D7 have definitive diagnoses of Verheij syndrome, and BG62 has a provisional diagnosis of Verheij syndrome. Subject D7 has been previously published [60]. Four additional individuals with *PUF60* pathogenic variants and TOF have been described in the literature [60–63]. IMPC data suggest that *Puf60*-null mice die prior to weaning of unknown causes but have not been described as having CHD [56]. These findings, combined with *PUF60’s* positive TOF-specific rank annotation score (79.9%), suggest that pathogenic variants in *PUF60* can cause TOF as part of Verheij syndrome.

#### *MEIS2*

Heterozygous loss-of-function variants in *MEIS2* are associated with *MEIS2*-related syndrome which presents with cleft palate, cardiac defects, and impaired intellectual development (MRS, MIM# 600987) [64]. Cardiac defects associated with this condition typically involve septal defects and aortic coarctation. *MEIS2* has also been proposed as the candidate gene causing cardiac defects associated with 15q14 deletions which have been documented in some individuals with TOF [65, 66]. In our cohort, BG31 and BG26 have probable and provisional diagnoses of MRS, respectively. Verheije et al. also describe an 18-year-old female with TOF and Ebstein’s anomaly who harbored a de novo pathogenic c.383delA, p.(Lys128Serfs*19) [NM_170675.5] frameshift variant in *MEIS2* [66]. We also note that *MEIS2* is essential for cardiac neural crest development, which is required for normal heart outflow tract formation as demonstrated in *Meis2*-null mice models which die perinatally with persistent truncus arteriosus and absent heart valves [67]. These findings, and *MEIS2*’s positive TOF-specific rank annotation score (61.9%), suggest that individuals with deleterious variants in *MEIS2* can present with TOF in the setting of MRS.

### Clinical practice recommendations

Since the diagnostic yield of CMA in individuals with TOF has been reported to be between 10–20%, with 22q11.2 deletion syndrome alone having a prevalence of approximately 10% in individuals with TOF, it seems reasonable to consider cES testing in individuals with TOF+ if CMA fails to identify a cause [7, 12].

Although gene panel testing is typically less expensive than cES, the difference in price varies based on a variety of factors including insurance coverage and the laboratories performing each test. In general, the higher cost of cES must be balanced against its higher diagnostic yield and the potential benefits of making a molecular diagnosis. These benefits may include the development of individualized medical care plans, access to emerging therapies, accurate genetic counseling, improved prognostication, psychological relief, and increased levels of social and emotional support through engagement with those who share the molecular diagnosis [68]. Families and society may also benefit economically through the avoidance of medical waste in the form of unnecessary testing, imaging, and/or medical procedures. As an alternative to CMA and cES, clinical genome sequencing (cGS), with its ability to detect both sequencing variants and CNVs, could be considered as a stand-alone test for individuals with TOF + .

Our data also suggest that further testing to identify an independent cause for TOF in individuals with genetic syndromes caused by pathogenic variants in *DVL3, MED13L, PUF60*, and *MEIS2* is not warranted.

## Supplementary information

Supplemental Table S1

Supplemental Table S2

Supplemental Table S3

Supplemental Table S4

## Data Availability

The data generated during this study can be found within the published article and its supplementary files. All variants reported here have been submitted to the ClinVar database (https://www.ncbi.nlm.nih.gov/clinvar/).
